# Direct pyrolysis of catalysed raw sewage sludge unlocks superior energy recovery: a kinetic evidence

**DOI:** 10.1007/s11356-026-37547-9

**Published:** 2026-02-26

**Authors:** Alberto Palma López, Susana Lozano Calvo, Mercedes Ruiz-Montoya, Manuel Jesús Díaz-Blanco

**Affiliations:** Chemical Engineering Dept., Universidad de Huelva, Huelva, Spain

**Keywords:** Sewage sludge, Catalytic pyrolysis, Activation energy, Flynn–Wall–Ozawa method

## Abstract

The present study investigates the pyrolysis kinetics of raw (undigested) sewage sludge and evaluates the effect of three low-cost catalysts (CaO, MgO, and Al_2_O_3_). The activation energies (Ea) were determined by means of the Flynn–Wall–Ozawa method. The raw sludge displayed a three-phase Ea profile, which suggests sequential decomposition of heterogeneous biopolymers. Among the catalysts, CaO exhibited the most significant reduction in Ea within the medium conversion range (*α* ~ 0.4–0.7). The effect of MgO was found to be reaction stage-dependent, while Al_2_O_3_ was observed to increase Ea in the initial stages, thus potentially promoting condensation reactions. The findings provide a robust foundation for the direct pyrolysis of raw sludge with low-cost catalysts, a process that has been demonstrated to enhance energy recovery in a single step, thereby circumventing the losses that are typically associated with prior anaerobic digestion.

## Introduction

The global increase in the production of sewage sludge, the primary by-product of wastewater treatment, poses substantial environmental and economic challenges in terms of its recovery and disposal (US EPA 2025). Current sludge management strategies must navigate the conflict of environmental regulations, economic costs, and public acceptance issues. Among conventional disposal methods, the use of agricultural land as a final disposal site remains the most controversial due to the potential transfer of heavy metals, microplastics, and persistent organic pollutants into ecosystems (US EPA 2025). Consequently, there is an increasing demand for alternative recovery technologies that are compatible with the principles of the circular economy and the United Nations Sustainable Development Goals, particularly SDG 7 (Affordable and Clean Energy).

A fundamental difference in sludge management lies in the distinction between non-digested (raw) sludge and sludge that has been anaerobically digested (AD). Raw sludge is characterised by high volatile matter (VM) content and reactivity. In contrast, digested sludge undergoes anaerobic digestion (AD), a process that produces biogas but reduces its VM and stabilises its organic content, resulting in a material richer in inert ash (Li et al. 2025). In Europe, the most common pathway is the two-stage process of AD followed by agricultural use (Pérez-García et al. 2025). However, this process does not eliminate the need for final disposal, because it merely reduces the sludge into a digested biosolid that still requires management. Additionally, the high moisture content of both sludge types poses a universal technical and economic obstacle.

In this sense, thermochemical methods represent a suitable alternative for energy recovery from sewage sludge. Pyrolysis, the thermal decomposition of organic matter in the absence of oxygen, is a particularly promising process because of its ability to immobilise heavy metals and destroy organic contaminants (Zhang et al. 2025). While the direct use of undigested sludge via thermochemical routes has received limited attention, it is warranted from an energy perspective. However, AD consumes up to 50% of the sludge’s total energy potential to produce biogas (Aragón-Briceño et al. 2021), a lower-value product than the versatile syngas obtained from the pyrolysis of raw sludge. Direct pyrolysis of raw sludge allows for the concentration and maximisation of biomass energy within a single conversion process.

The use of low-cost catalysts is a key area of process optimization to reduce the energy costs associated with the high activation energies of pyrolytic degradation. These catalysts have been shown to reduce reaction temperatures and kinetics, while also minimising the release of nitrogenous pollutants such as NH_3_ and HCN (Csutoras and Miskolczi 2024). It is imperative to understand these kinetic parameters and catalyst effectiveness in order to optimise reactor design and enhance process efficiency. The characteristics and pyrolytic kinetics of raw sewage sludge were investigated using various low-cost catalysts. This will allow us to elucidate the degradation and energy requirements during pyrolysis. The results of this study will provide data for developing efficient, raw material-specific pathways for energy recovery from undigested sludge.

## Materials and methods

### Characteristics of raw materials

Sewage sludge was extracted from a municipal wastewater treatment plant in Huelva (Spain) operated by the municipal company Aguas de Huelva. The activated sludge process was used to treat wastewater in this plant. The non-digested sludge or raw sludge (RS) was directly taken from the decanting after the process. The digested sludge (DS) studied was obtained from the digestion process and subsequent dewatering by filter-press. Both sludge types dried in an oven at 70 °C for 48 h. After the drying process, the sludge was crushed, screened, and packed in vacuum plastic bags. Table 1 reports the characteristics of the raw materials.

**Table 1:** Sewage sludge composition (o.d.b.)

	Typical raw sewage sludge (Zarina and Mezule (2023); Villalobos-Delgado et al. (2023); Skripsts et al. (2024))	Present study Raw sewage sludge	Typical digested sewage sludge (Jimenez et al. (2013); Liu and Smith (2022); Lu et al. (2024))	Present study Digested sewage sludge
Proximate analysis				
Organic matter (%)	57–82	77.2	64–73	50.8
Proteins (%)	18–35	29.1	13–27	17.5
Lipids (%)	8–22	17.5	5–12	8.3
Carbohydrates (%)	11–25	20.7	5–24	12.9
Ash (%)	15–25	17.6	23–45	23.5
Ultimate analysis^a^				
C (%)	35–45	27.9	27–41	33.9
H (%)	5–11	4.7	5–9	4.2
N (%)	4–6	4.5	3–5	4.7
O^b^ (%)	38–63	34.6	42–56	51.2
P (%)	1–3	2.5	1–2	1.9
S (%)	0.5–1.5	1.4	0.5–1.0	0.6

^a^Ultimate analysis was performed using Carlo Erba 1108

^b^Oxygen (% wt) = 100—carbon (%)—hydrogen (%)—nitrogen (%)—sulphur (%)—other elements contained in the ash (%)

### Selected catalysts

The catalysts examined in this study are metal oxides (Al_2_O_3_, MgO, and CaO), which were chosen for their cost-effectiveness, despite presenting certain disadvantages compared to higher-cost alternatives such as lower resistance to deactivation and lower selectivity and activity (Zhang et al. 2015).

Alumina (Al_2_O_3_) is a catalyst that is described as having high catalytic activity due to its porous structure. It demonstrates excellent thermal stability, with a maximum operating temperature of 800 °C, and a high adsorption capacity and the presence of Lewis acid sites in alumina could facilitate the breaking of C-O bonds, thereby promoting deoxygenation and enhancing the selectivity of valuable hydrocarbons (Oh et al. 2022). Magnesium oxide (MgO) is produced by calcining magnesium carbonate and its cost-effectiveness, coupled with its notable resilience to deactivation from biomass ash and alkali metals, makes it a highly attractive solution (Stefanidis et al. 2016). Calcium oxide (CaO) is also considered to have high catalytic activity, low cost, and environmental friendliness. In addition, its high basicity gives it deacidifying properties (Hakim et al. 2024). The catalysts were activated by calcining them in a muffle furnace at a constant temperature of 700 °C for 5 h while they were kept in a ceramic container.

### Analytical methods

Moisture content of the sludges have been determined by ASTM D2216-98 (1985), oven drying at 110 °C to constant weight. Moreover, ASTM E1755-0128 was adopted to determine the ash content in the selected sewage sludge; in this form, 2 g sample in a standard crucible was pre-weighed and then incinerated in a muffle furnace at 760 °C for 2 h (greyish white matter obtained). After cooling in a desiccator, it was reweighed. The ash content is calculated by Eq. (1).

1 $$Ash \,\left(\%\right)= \frac{{W}_{1} - {W}_{c}}{{W}_{2} - {W}_{c}} \times 100$$

where W_c_ is the weight of the crucible; W_1_ is the weight of the ash and the crucible; W_2_ is the weight of the sample and crucible.

ASTM E872-8229 has been followed to obtain the volatile matter of the biomass. A 2-g sample of each biomass was heated in a standard crucible in a muffle furnace at 800 °C for 7 min. Subsequently, it was cooled in a standard desiccator and reweighed. The volatile matter was determined by Eq. (2).

2 $$\text{Volatile Matter }(\%) = \frac{{W}_{1} - {W}_{2}}{{W}_{1}} \times 100$$

where W_1_= previous weight of the sample; W_2_ = sample final weight after incineration.

EPA 3105 A standard test has been used for heavy metal determination. A sludge sample (1g) was dissolved in concentrated nitric acid using microwave heating microwave-assisted acid digestion method. The heavy metal content of the digestate was then measured with Inductively Coupled Plasma Optical Emission Spectroscopy (ICP‑OES) (ICP-MS Agilent 7700). No glassware was used for digestion and dilution.

### Thermogravimetric analysis

Thermogravimetric experiments were performed for each of the thermochemical treatments, and these were carried out in the Mettler Toledo TGA/DSC1 STARe System. Following the process of homogenisation, the samples of ground and well materials have been distributed into various alumina crucibles. Furthermore, the samples are automatically introduced into the oven by a robot. In the pyrolysis process, nitrogen (30 mL mN^−1^) was used as an inert gas to prevent any form of the secondary reaction.

The thermochemical processes of the samples were conducted in three steps: The initial stage of the experiment involved subjecting the sample to heating from 25 to 105 °C at a rate of 15 °C min^−1^. This stage was conducted in an inert nitrogen atmosphere at a flow rate of 30 mL min^−1^. The temperature was then maintained at 105 °C for a duration of 5 min, with the flow and atmosphere being sustained from the first stage to ensure the complete loss of moisture from the sample. The subsequent stage involved heating from 105 to 800 °C at various heating rates, of 5, 10, 15, 20, and 25 °C min^−1^. In all cases, the initial sample mass was within the range of 15–20 mg. Experiments were performed in triplicate and the mean values were recorded.

### Flynn-Wall-Ozawa kinetic method

The Flynn-Wall-Ozawa (FWO) method, a widely used isoconversional approach for kinetic analysis of biomass pyrolysis, originates directly from the differential form of the Arrhenius equation, enabling the determination of activation energy as a function of conversion without presuming a reaction model.

The fundamental equation of the (FWO) method is expressed as Eq. (3) (Ozawa 1965; Flynn and Wall 1966).

3 $$log\,(\beta)=log\,(AE/Rg\,(\alpha)-0.4567\,E/RT$$

where *β* denotes the heating rate, *A* is the pre-exponential factor, *E* is the activation energy, *R* is the gas constant, *T* is the absolute temperature, *g*(*α*) is an integral conversion function, and *α* represents conversion degree.

Therefore, this equation can be used to estimate the kinetic parameters from thermogravimetric data at varying heating rates.

The primary benefit of the FWO method is its model-free nature, which eliminates the need to assume a specific reaction pathway. This characteristic ensures that robust kinetic parameters for complex materials and reaction stages. Thus, FWO is regarded as a highly reliable method for non-isothermal thermogravimetric analysis of heterogeneous feedstocks such as sewage sludges (Guo et al. 2025) and non-isothermal feedstocks. This process is highly efficient, minimising experimental error and revealing variations in activation energies over different conversion degrees.

Therefore, the proposed model could be an excellent basis for initial screening and comparison (Sarkar and Wang 2020). The NETZSCH Kinetics Neo Professional Edition (v. 2.4.4.6) was used to evaluate the kinetic parameters of the samples.

## Results and discussion

### Compositional differences of raw materials

The composition of the raw and digested sewage sludge evaluated in this study (Table 1) shows data similar to that found within the typical ranges reported in scientific literature for most parameters. In this regard, both raw and digested sludge show values that fall within typical ranges, indicating that the sample is representative of conventional sludge. However, a detailed elemental analysis reveals certain discrepancies, particularly in the carbon content (27.9%), which is below the typical range of 35–45%, and the oxygen content (34.6%), which is also below the range of 38–63%. These atypical values could be related to the influence of specific industrial discharges or to a higher proportion of inert material in the analysed sample.

When comparing the digested sludge in this study with typical ranges, the proximate components are in line with what is expected after AD. The organic matter has decreased to 50.8%, which is within the range of 44–63%. This result is consistent with the biodegradation of volatile organic matter, as shown by the reductions in proteins, lipids, and carbohydrates. The increase in ash content (23.5%) is consistent with the range reported in the literature, because of the concentration of inorganic fractions after microbial degradation. With regard to elemental analysis, the nitrogen content (4.7%) is slightly above the typical range of 3–5%, which could be due to nitrogen retention in ammoniacal forms or the composition of the residual microbial biomass. In contrast, hydrogen (4.2%) is below the range of 5–9%, possibly due to the preferential degradation of hydrogenated compounds during digestion, as documented in previous studies on sludge stabilisation (Xiao et al. 2020; Sailer et al. 2020).

In addition, the comparison between raw and digested sludge indicates compositional changes, which are expected as a result of AD. In this regard, there is a marked decrease in organic matter, as well as in proteins, lipids, and carbohydrates, reflecting the conversion of these substrates into biogas and other metabolic products. Conversely, the ash content increased, which is consistent with the non-biodegradable material concentration. The elemental analysis also shows an increase in the percentage of carbon in raw sludge compared to digested sludge, which can be explained by the selective degradation of other components and the relative stability of certain carbonaceous fractions (Hao et al. 2020; Liu and Smith 2022). Similarly, the increase in oxygen content (from 34.6 to 51.2%) suggests the formation of oxidised compounds, while the reduction in sulphur may indicate its volatilisation or precipitation during treatment (Kang et al. 2022; Zhang et al. 2023).

### Thermogravimetric analysis of raw and digested sewage sludge

**Fig. 1:**
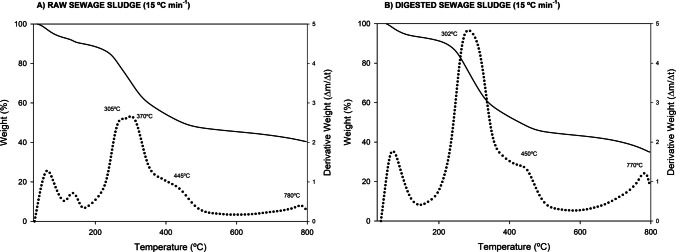
Thermogravimetric (TGA) and derivative thermogravimetric (DTG) evolution of raw (non digested) (**A**) and digested (**B**) sewage sludge

Thermogravimetric (TGA) and derivative thermogravimetric (DTG) analyses reveal distinct pyrolysis degradation profiles for raw and anaerobically digested sewage sludge, highlighting the significant impact of each composition and the AD process (Fig. 1). The initial mass loss below 150 °C, which is attributable to moisture evaporation, was observed in both materials. The primary devolatilization of organic matter occurred within the 200–600°C range, a finding consistent with that of other studies (Vali et al. 2025). However, the principal distinction, however, lies in the characteristics of the DTG peaks within this range. The raw sludge exhibited a broader DTG peak of lower intensity, aligning with the observations made by Zhang et al. (2025). Digested sludge demonstrates a more intense and narrow decomposition peak, typically centred around 300°C. This prominent peak is indicative of the rapid and concurrent degradation of readily biodegradable components, such as proteins, carbohydrates, and lipids, indicating a higher volatile matter content (Petrovič et al. 2021). This profile is the result of the consumption of the most labile organic fractions during AD, leaving behind a more stable organic matrix. Further critical differentiation was evident at higher temperatures (>500°C). Digested sludge exhibits a more significant mass loss in this region, which can be attributed to the decomposition of this stabilised residual organic matter or the decarboxylation of inorganic carbonates (Petrovič et al. 2021). A comparison of the two evolution profiles reveals similarities in the general TGA/DTG profiles, indicating fundamentally similar thermal degradation mechanisms. However, the primary distinction lies in the extent of mass loss. In this regard, raw sludge demonstrates a greater degree of mass loss in the primary degradation region (~300°C) due to its higher volatile matter content. Consequently, digested sludge exhibits lower mass loss in this range and a slightly higher final residue, as a result of the stabilisation of organic matter and the relative concentration of inert materials during the AD process (Zhang et al. 2025). These fundamental differences in reactivity and composition directly govern pyrolysis kinetics and ultimately determine biochar yields.

### Kinetic analysis of raw sewage sludges using catalisers

**Fig. 2:**
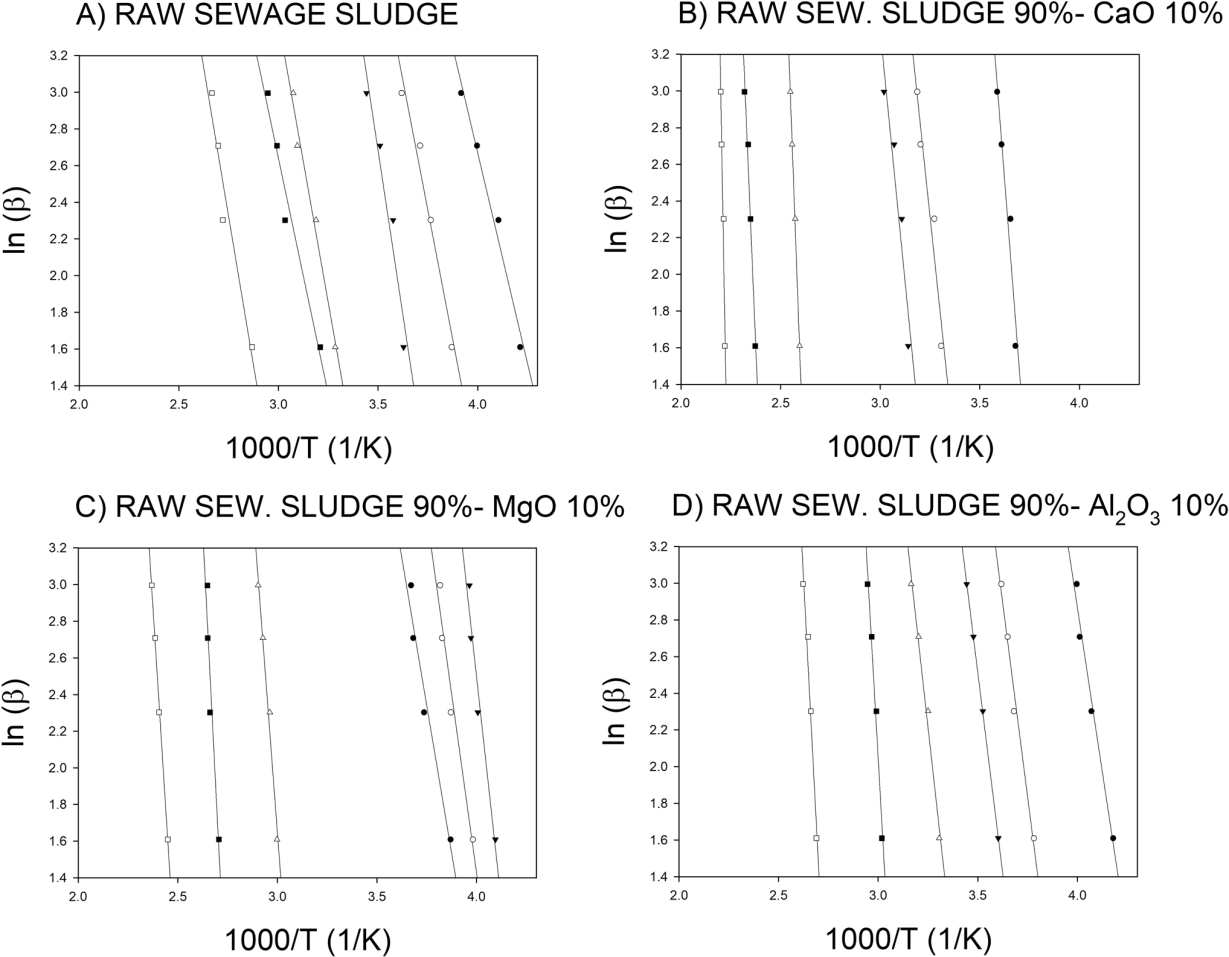
Arrhenius plot of ln(*β*) vs. 1000/T (from *α* = 0.3 to 0.8) for the selected biomass-to-catalyst ratio under different thermochemical processes (5–20 °C min^−1^)

The plots of 1000/T vs. log (*β*) are shown for the pyrolytic degradation of raw sewage sludge (Fig. 2A) and its combination with the selected catalysed CaO (Fig. 2B), MgO (Fig. 2C), and Al_2_O_3_ (Fig. 2D), respectively, using the FWO model. The plots of the used model show adequate determination coefficients (R^2^>0.91) obtained from the plots of the used model. As described in Eq. (3), the activation energy for the pyrolysis of each sample was determined from the slope of the straight line for the plots of log (*β*) and 1000/T.

The consistently high *R*^2^ values confirm the efficacy of the FWO method for these systems. However, it should be noted that linearity does not necessarily imply a single reaction mechanism, and sewage sludge is a complex matrix with components that decompose in overlapping temperature ranges. Therefore, the derived Ea value is an average value that fits several consecutive or parallel processes. The addition of catalysts is likely to alter the dominant reaction pathway.

As illustrated in Fig. 2, the introduction of basic oxides, specifically CaO and MgO, has a significant impact on the kinetics of the process when compared to raw sewage sludge. This is evident in the difference between the slopes of the straight lines in Fig. 2B and C, which differ from those corresponding to raw sludge. This phenomenon can be attributed to the catalytic activity of these oxides, which can promote cracking and reforming reactions in the solid phase, facilitating the breaking of macromolecular bonds present in the biomass (Wang et al. 2022, 2024).

In contrast, the behaviour of Al_2_O_3_ (Fig 2D) appears to be less pronounced. Its amphoteric nature and Lewis acid site structure offer a different catalytic mechanism, often associated with dehydration and recombination reactions (Fernandez et al. 2021). These do not always lead to a significant decrease in the overall Ea for this specific type of matrix.

In this regard, authors such as Fernandez et al. (2021) and Guo and Wang (2023) demonstrated that MgO was more effective than alumina in reducing Ea in the pyrolysis of agroforestry residues, which is consistent with the trend shown in this study.

**Fig. 3:**
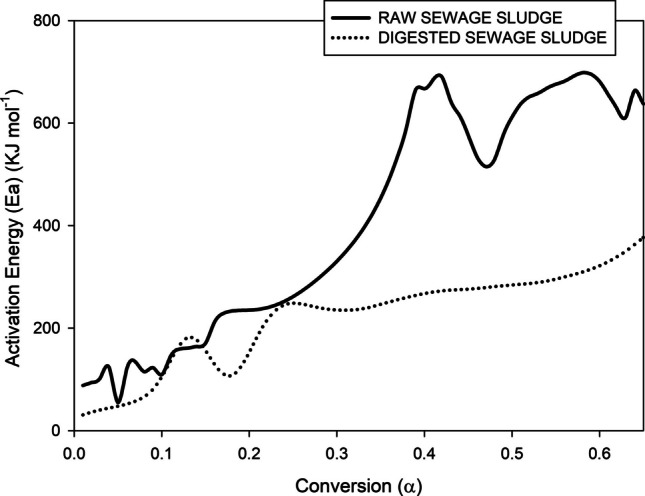
Evolution of the activation energy of both sewage sludges

As shown in Fig. 2, the selected kinetic model is highly suitable for simulating the pyrolytic degradation of sludge, in the absence and presence of additives and with different catalysts. Therefore, the activation energy evolution of both types of sludge must be compared to support the addition of catalysts to raw sewage sludge (Fig. 3).

The thermal decomposition of both sewage sludges (Fig. 3) exhibits distinct kinetic profiles that are fundamentally altered by the AD process. A comparative analysis of the evolution of activation energy (Ea) during pyrolysis as a function of conversion (*α*) reveals a multi-stage mechanism for non-digested sludge and a more uniform mechanism for the digested sludge, directly attributable to the biochemical stabilisation of the organic matrix.

In this form, the pyrolysis of non-digested sludge is characterised basically by a tri-phasic Ea profile, which indicates a complex mixture of organic components. The initial phase (low *α*, ~0.1–0.3) shows a high and often decreasing Ea, which could correspond to the primary devolatilization of labile biopolymers such as proteins, carbohydrates, and lipids (Gao et al. 2020). Although these compounds have varying thermal stabilities, they are generally more reactive than residual solids. The second phase (mid *α*, ~0.4–0.7) typically presents a lower and relatively stable maximum Ea, representing the co-combustion of the remaining organic matter and the onset of the decomposition of recalcitrant substances (Urban and Antal 1982). The final phase (high *α*, >0.7) is marked by a narrow, significant decrease in Ea. This is a characteristic of the pyrolysis of a stable carbonaceous residue, which involves the breakdown of aromatic structures, polycondensed carbon, and the initiation of secondary char-forming reactions (Font et al. 2005).

In contrast, the digested sludge displayed a near-monophasic profile, characterised by a consistently low and stable Ea across most of the conversion range (*α* ~0.1–0.8), followed by a moderate increase at the terminal conversion. This simplified kinetic behaviour is a direct consequence of the AD, which acts as a biological pretreatment. During digestion, methanogenic microorganisms preferentially consume the same labile, volatile organic fractions (proteins, carbohydrates) that drive the complex, multiphasic profile in the raw sludge. Gao et al. (2014) noted that this consumption leaves a residual solid matrix enriched in recalcitrant compounds, such as microbial biomass (bacterial cells), lignin, and inert materials. The resulting feedstock is more chemically homogeneous and thermally stable, leading to a pyrolysis process dominated by the decomposition of this recalcitrant pool, hence the flatter Ea profile. The final moderate increase in Ea is associated with the degradation of the most stable carbonaceous components, but its lesser magnitude compared to non-digested sludge suggests a different char structure, potentially with a lower complex aromaticity due to the altered initial composition.

### Kinetic effects of adding catalysts to raw sewage sludge

**Fig. 4:**
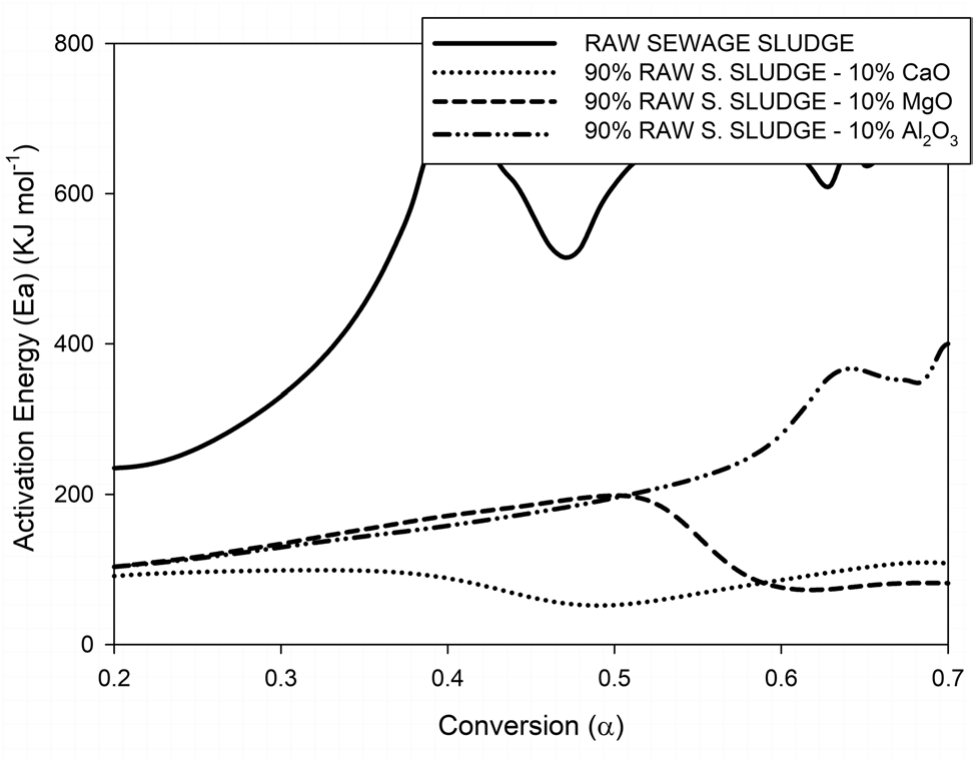
Comparative evolution of the activation energy of crude sludge with and without different catalysts

Figure 4 presents a comparison of the activation energy of raw sewage sludge and sludge containing catalysts.

The activation energy (Ea) of raw sewage sludge decreases significantly after the incorporation of 10% CaO, particularly in the medium conversion range, compared with the material without additives. This reduction can be attributed to the basic nature of CaO, which catalyses cracking reactions by facilitating proton transfer and the breaking of C-C bonds in the organic components of the sludge (Li et al. 2024). Calcium oxide acts as an agent capable of modifying the reaction pathway, decreasing the activation energy throughout the sludge degradation interval.

The Ea evolution with MgO shows slight deviation. In this instance, the observed decrease is less marked than that observed with CaO at low *α* values, but becomes more evident at higher conversions. This profile could indicate a catalytic mechanism that is strongly dependent on the progress of the reaction and the degradation of the components in each phase. As El Bari et al. (2024) previously established, the moderate basic capacity of MgO, in conjunction with its specific crystalline structure, exerts a significant influence on the sequential decomposition of lignocellulosic components, including cellulose, hemicellulose, and lignin. These components exhibit divergent energy requirements and varying levels of affinity for the catalyst.

Similar to the results reported by Zhu et al. (2024), the addition of Al_2_O_3_ produces a different Ea evolution curve. In this case, the activation energy in the initial stages may exceed that of the raw sludge and then decrease at high *α* values. Migliorero et al. (2025) suggested that this phenomenon could indicate the presence of alumina, an acidic substance. Alumina can promote condensation or dehydration reactions. These reactions require additional energy input and subsequently favour the gasification of the formed carbonaceous residues. Moreover, de Van Minkelis et al. (2025) indicated that the porous texture of the catalyst can also affect the diffusion of volatile compounds, thereby modifying the overall kinetics.

## Conclusions

The impact of anaerobic digestion on the pyrolysis kinetics of sewage sludge has been found to be a significant factor in the study. Digested sludge has been shown to exhibit a significantly more stable and monophasic activation energy (Ea) profile as a function of conversion, as a result of the prior biological removal of the most labile organic fractions. Conversely, raw sludge exhibits a complex three-phase profile, indicative of the sequential decomposition of a heterogeneous mixture of biopolymers.

In the evaluation of low-cost catalysts, CaO has been shown to be the most efficient in reducing the activation energy required for the pyrolysis of raw sludge, particularly within the medium conversion range (*α* ~0.4–0.7). This reduction is attributed to its high basicity, which catalyses homogeneous cracking reactions and neutralises intermediate acids, thus modifying the overall reaction pathway in a favourable manner. MgO has been shown to reduce Ea, albeit with a less pronounced catalytic effect at low conversions and a greater dependency on the reaction stage. It appears that the mechanism is linked to the sequential decomposition of lignocellulosic components. Conversely, Al_2_O_3_, being acidic, has the potential to enhance the Ea in the initial stages, likely by encouraging condensation reactions, prior to facilitating the gasification of carbonaceous residue at high conversions.

The study confirms that direct pyrolysis of raw sludge, assisted by basic catalysts such as CaO, is a viable technical route to maximise energy recovery in a single process, avoiding the loss of energy potential associated with prior AD.

## Data Availability

Bulk data are available in 10.5281/zenodo.17955788.
